# A double-blind, randomized, placebo-controlled study assessing the impact of probiotic supplementation on antibiotic induced changes in the gut microbiome

**DOI:** 10.3389/frmbi.2024.1359580

**Published:** 2024-03-22

**Authors:** Daniel John, Daryn Michael, Maya Dabcheva, Eleri Hulme, Julio Illanes, Tom Webberley, Duolao Wang, Sue Plummer

**Affiliations:** ^1^ Research & Development, Cultech Ltd, Port Talbot, United Kingdom; ^2^ Clinical Research Unit, Comac Medical, Sofia, Bulgaria; ^3^ Department of Clinical Sciences, Liverpool School of Tropical Medicine, Liverpool, United Kingdom

**Keywords:** probiotics, microbiome, antibiotics, antibiotic resistance, metagenomics

## Abstract

The human gut microbiome, crucial for health, can be disrupted by antibiotic treatment, leading to various health issues and the rise of antimicrobial resistance (AMR). This study investigates the impact of a probiotic on the gut microbiome’s composition and antimicrobial resistance genes (ARGs) content following antibiotic treatment. Conducted as a single-centre, double-blind, randomized, placebo-controlled trial, adults taking oral antibiotics were allocated into a probiotic or placebo group. Evaluations included viable cell enumeration and shotgun metagenomic sequencing for microbiome analysis, along with ARG assessment. The probiotic maintained the numbers of lactobacilli, significantly increased the Bacteroides population and decreased numbers of enterobacteria. The lactobacilli and enterococci numbers decreased in the placebo. The alpha diversity remained stable in the probiotic group throughout the study, but significant reductions were observed in the placebo group post antibiotic treatment. There was significant spatial separation in beta diversities between groups at the end of the study. Compared to baseline levels, there was a significant reduction in the abundance of ARGs in the probiotic group at the end of the study, while ARG abundance in the placebo group was comparable with baseline levels at the end of the study. Co-occurrence network analysis observed consistent betweenness centrality and node degree within group in the probiotic group whereas scores decreased in the placebo group. This study suggests that the probiotic may minimize the disruption of antibiotic treatment on the gut microbiome by preserving microbial diversity and reducing ARG abundance.

## 1 Introduction

The gut microbiome is a complex community of microorganisms residing in the gastrointestinal (GI) tract, which plays a key role in human health, influencing metabolism, immunity, and behaviour (Dinan and Cryan, 2017; Zheng et al., 2020; Fan and Pedersen, 2021). Disturbances in the gut microbiome induced through antibiotic treatment can negatively affect overall diversity, potentially allowing harmful bacteria to become dominant or even eradication of certain species (Yang et al., 2021; Patangia et al., 2022). This can lead to disruption of the balance of the gut microbiome and imbalances have been linked to conditions such as obesity, inflammatory bowel disease, type 2 diabetes and colorectal cancer (Cuevas-Sierra et al., 2019; Artemev et al., 2022; Zhou et al., 2022; Kim et al., 2023). It has been shown that the balance of the gut microbiome of healthy adults can recover following antibiotic exposure, however, repeated exposures can significantly extend recovery time and risk permanent disruption within the microbiome (Anthony et al., 2022).

Antimicrobial resistance (AMR) presents a significant and growing threat to public health and is currently responsible for an annual death toll of approx. 700,000 people worldwide. This threat is projected to rise to around 10 million deaths by 2050 (Murray et al., 2022). Antibiotic treatment has been shown to deplete indigenous gut bacteria while increasing the antibiotic resistant gene (ARG) pool, creating a reservoir of ARGs within the gut microbiome, known as the gut resistome (Elvers et al., 2020; Ramirez et al., 2020; Crits-Christoph et al., 2022; Patangia et al., 2022). The ARGs from commensal organisms can be disseminated through mobile genetic elements (MGEs) or via bacteriophage transduction to various species, including potential pathogens, through horizontal gene transfer (HGT) (Lerner et al., 2017; McInnes et al., 2020).

Probiotics are defined by the World Health Organization as “live microorganisms which when administered in adequate amounts confer a health benefit on the host” (Hill et al., 2014). The interactions between probiotics and the gut microbiome remains an emerging area of research with potential benefits (Wang et al., 2021; Vijay and Valdes, 2022), although currently, it is unclear the effect probiotics have on modulation of the gut microbiome and resistome during and post antibiotic treatment, with studies showing varying effects (Éliás et al., 2023). This study aimed to investigate the effect of a probiotic formulation, comprising *Lactobacillus acidophilus*, *Bifidobacterium bifidum*, *Bifidobacterium animalis* subsp. *lactis* and *Saccharomyces boulardii*, on the composition of the gut microbiome following antibiotic treatment.

## 2 Materials and methods

### 2.1 Study approval

The study was conducted in accordance with the ethical principles of the Declaration of Helsinki and ethical approval was granted by the Ethical Committee of Comac Medical, Sofia, Bulgaria (Reference: #246/13/07/2022). The study protocol was registered with clinicalTrials.gov on 19/04/2022: NCT05355571.

### 2.2 Study design

A single-centre, double blind, randomized, placebo-controlled study with equal allocation of participants between two parallel study groups was undertaken. Adults (aged 18–65) who had been prescribed an oral antibiotic for non-gastrointestinal disturbance were recruited by Comac Medical (Sofia, Bulgaria). Physicians, located in Sofia, Bulgaria, prescribing the antibiotics offered potential candidates the opportunity to take part in the study. The inclusion criteria were: receiving a 5 to 10-day course of oral antibiotics for a non-GI related condition, refraining from any other probiotic or prebiotic supplements during the study, willing to maintain normal lifestyle and diet throughout the study, willing to refrain from taking any non-GP prescribed antibiotics during the study and willing to provide faecal samples. Exclusion criteria included: antibiotic intake within the previous three months, regular probiotic intake during the month prior to the study, immunodeficient or undergoing immunosuppressive therapy, pregnancy or planning pregnancy, diabetes, having cardiovascular disease or severe systemic disease.

A total of 50 candidates were recruited in November 2022, with the study taking place between 18/11/2022 and 17/01/2023; there were no dropouts or adverse effects reported in either arm of the study. Baseline demographics of the populations are shown in **Table 1**. The oral antibiotics used during the study included amoxicillin, cephalosporins, azithromycin, clarithromycin, clindamycin and spiramycin. The average antibiotic course length was 5.76 days in the placebo group, and 5.92 days in the probiotic group.

**Table 1:** Baseline demographics and characteristics.

	Placebo	Probiotic	p-value
Baseline demographics			
No of participants	25	25	
Male (%)	40	56	0.8512
Female (%)	60	44	
Age, years (SD)	47.64(11.7)	45.44(11.5)	0.5039
Height, m (SD)	168.92(9.3)	172.84(9.8)	0.1576
Weight, kg (SD)	78.028(13.7)	75.096(13.5)	0.4485
BMI, kg/m^2^ (SD)	27.36(4.2)	25.02(4.1)	0.4123
SBP, mmHg (SD)	125.6(9.2)	125.44(9.02)	0.9508
DBP, mmHg (SD)	78(6.3)	78.52(6.2)	0.7698
Antibiotic class			0.8539
β-Lactams	13	14	
Macrolides	12	11	
Antibiotic			0.6398
Amoxicillin	5	5	
Cephalosporins	8	9	
Azithromycin	5	7	
Clarithromycin	6	3	
Clindamycin	0	1	
Spiramycin	1	0	
Antibiotic course length (Days)			0.6516
5	15	14	
6	4	3	
7	5	6	
8	0	1	
9	0	0	
10	1	1	

### 2.3 Randomization

The eligible participants were allocated to one of the two study arms in a 1:1 ratio according to a computer-generated random sequence (block-size of four) that was generated using SAS PROC PLAN (SAS v9.4). The allocation sequence was not available to any member of the research team until all databases had been completed and locked. Tamper-proof sealed envelopes containing the participant allocation sequence were held at the trial site.

### 2.4 Study product

The probiotic product comprised capsules containing a total of 25 billion colony forming units (CFU) of *Lactobacillus acidophilus* CUL60 (National Collection of Industrial, Food and Marine Bacteria (NCIMB) 30157), *Lactobacillus acidophilus* CUL21 (NCIMB 30156), *Bifidobacterium bifidum* CUL20 (NCIMB 30153) and *Bifidobacterium animalis* subsp. *lactis* CUL34 (NCIMB 30172) and 10 billion CFU of *Saccharomyces boulardii* (Collection Nationale de Cultures de Microorganismes (CNCM)-I-1079) on a base of microcrystalline cellulose, silicon dioxide and magnesium stearate. The placebo only contained the base ingredients and was identical in appearance, size and weight to the probiotic product. The probiotic and placebo products were prepared by Cultech Ltd, Port Talbot, UK. One capsule of the probiotic or placebo was taken daily for 10 days. Participants were instructed to refrigerate the study product and take the capsules at least 2 hours after antibiotic ingestion.

### 2.5 Outcomes

**Figure 1:**
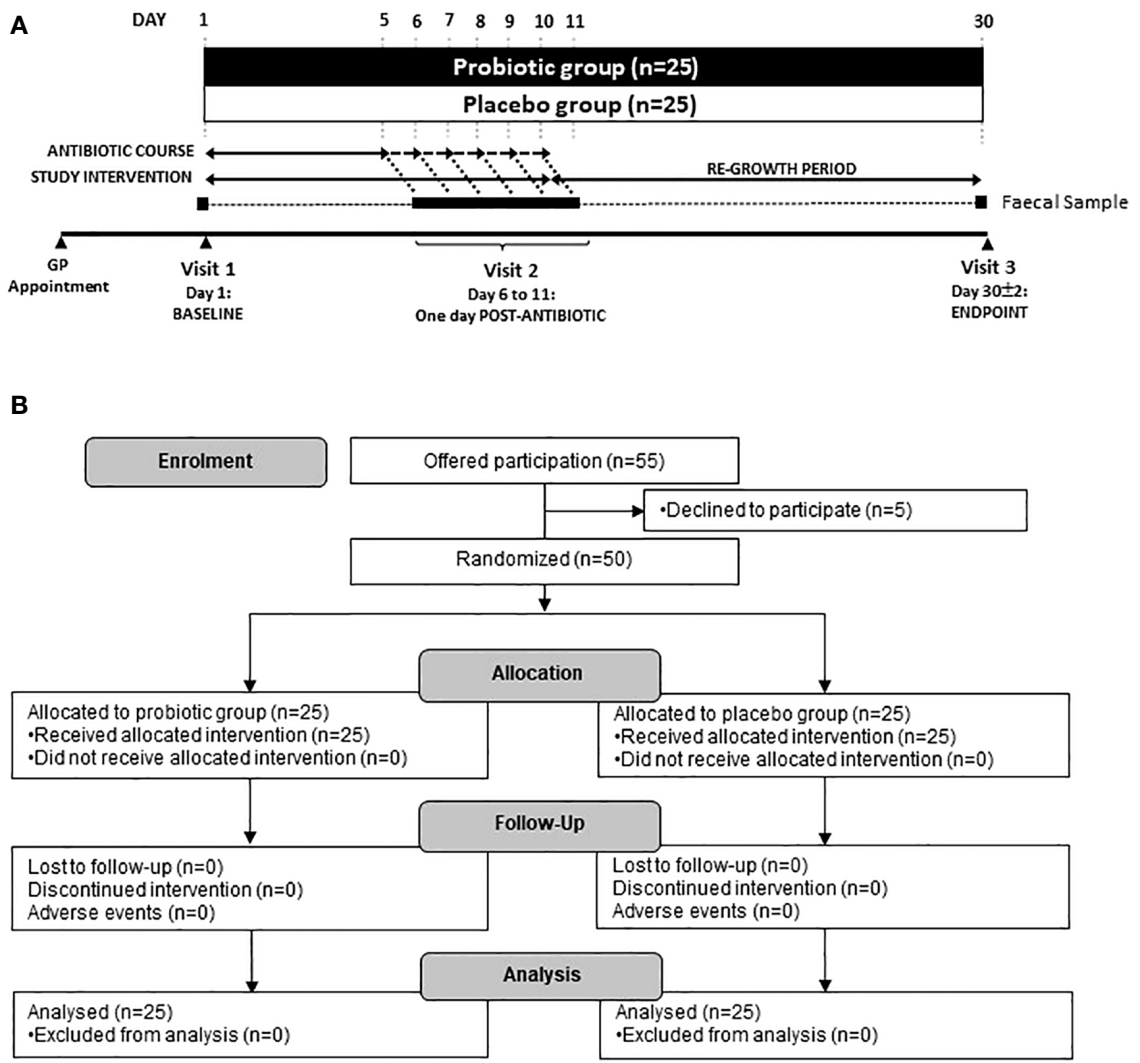
**(A)** Scheme of the study and sample collection and **(B)** Study flow diagram. Participants were recruited via their local physicians after being prescribed an antibiotic for 5–10 days. Three faecal samples were taken at Baseline (day 1), one day following the end of antibiotic treatment (day 6–11) and at the end of the study (day 30).

The primary study outcome was changes in the composition of the microbiome assessed through viable cell enumeration and shotgun metagenomic sequencing. Secondary outcomes were analysis of ARG content and abundance. The study overview of participant visits to the trial centre and sample collection is shown, along with a flow diagram of enrolment, allocation and follow-up in **Figure 1**.

### 2.6 Faecal sampling and collection

Three faecal samples were collected; the first on day 1 (baseline sample), the second after finishing the course of antibiotics (varying from days 6–11, post antibiotic sample) and the third at day 30 ± 2, (endpoint sample). Faecal samples were collected using the Fe-Col^®^ Faecal Sample collection kits (Alpha Laboratories, Hampshire, UK) and were transferred into anaerobic Genbags (Sigma Aldrich, UK). Full details of faecal collection procedure can be found in **Supplementary Figure S1**. Samples were stored at <10°C for up to 24 hours then stored at −80°C, pending analysis.

### 2.7 Bacterial enumeration and antibiotic sensitivity

Faecal samples were assessed for viable bacterial numbers using a modified version of the Miles Misra (1938) plate count technique. Decimal dilution series were set up in Maximum Recovery Diluent (MRD, Oxoid, UK) and were plated onto a range selective media (Oxoid, UK, **Supplementary Table S1**). Total Bacteria represents the sum of the counts from total aerobes and total anaerobes. Viable cell numbers were expressed as log_10_ of the number of CFU/g sample.

### 2.8 DNA extractions, metagenomic sequencing and quality analysis

DNA was extracted from faeces using the QIAamp^®^ Fast DNA Stool Mini Kit (Qiagen, Germany) as per the manufacturer’s instructions. The eluted genomic DNA samples were quantified using a Qubit^®^ (Thermo Fischer Scientific, Unite States) and stored at −20°C. Shotgun metagenomic sequencing was performed using the Illumina Novaseq 6000 platform (Novogene, China). Quality control of raw reads was performed using readfq V10 (https://github.com/cjfields/readfq) using default parameters. The specific processing steps were as follows: a) removal of reads comprising low quality bases (quality threshold value ≤38), >40 base pairs (bp); b) removal of reads wherein the N base reached 10 bp; c) removal of reads presenting an overlap >15 bp with Adapter. Host sequences decontamination was performed using Bowtie2 with the default parameters (Langmead and Salzberg, 2012).

### 2.9 Metagenomic data analysis

Host-filtered metagenomic samples were assembled using MEGAHIT (Li et al., 2015) and open reading frames (ORFs) in contigs were predicted using MetaGeneMark-2 (Gemayel et al., 2022). The predicted genes from each sample were merged and clustered using CD-HIT (Fu et al., 2012) based on the criteria of identity >95% and coverage > 90% to remove redundant genes. The gene abundance profiles were constructed using Bowtie2 and mapped to the Unigenes datasets. Annotation of ARGs was performed using the Resistance Gene Identifier (RGI) software provided by the Comprehensive Antibiotic Resistance Database (CARD 2023) (Alcock et al., 2023).

### 2.10 Statistical analysis

Baseline demographics and antibiotic usage of the groups were compared using either an unpaired two-way t-test (age, height, weight, Body Mass Index (BMI), Systolic Blood Pressure (SBP), Diastolic Blood Pressure (DBP) and antibiotic course length) or Fisher’s exact test (sex, antibiotic class and antibiotic type). Differences in the number of faecal CFU were assessed using a mixed-effects analysis with a Tukey’s *post-hoc* for multiple comparisons (GraphPad Prism, Version 10.0.2) where *p<0.05, **p<0.01 and ***p<0.001.

The R package Phyloseq (McMurdie and Holmes, 2013) was used for data importation and diversity analyses. A generalized linear model with antibiotic type and course length as covariates and *post-hoc* Bonferroni correction was employed using the R package emmeans (Lenth, 2024) to compare Shannon’s diversity index, changes in bacterial abundance and changes in ARG abundance between interventions and within timepoints. Spatial differences of the groups were observed with a Non-Metric Multidimensional Scaling (NMDS) plot based on Bray-Curtis dissimilarity matrix. The R package Vegan was used to perform permutational analysis of variance (PERMANOVA) computed (with 999 permutations) using the Adonis function and homogeneity of dispersion (Oksanen et al., 2022). Differential abundance analysis between interventions was analysed using DESeq2 (Love et al., 2014).

The co-occurrence network was generated based on the Spearman correlation matrix constructed with the WGCNA (weighted correlation network analysis) package (Langfelder and Horvath, 2008). The nodes in this network represent unique taxa at the genera level and the edges connecting these nodes represent correlations between taxa. All p-values were adjusted for multiple testing using the Benjamini and Hochberg false discovery rate (FDR) controlling procedure at a threshold of 0.05. To evaluate connectedness of the networks and highlight keystone genera, betweenness centrality was used to measure the proportion of the shortest paths in a network that pass through a node; a lower average betweenness centrality score represents a more connected network due to more short paths or fewer shortest paths through each node. Node degree was calculated as a measure of sparsity, with a lower degree equalling a sparser network. These metrics were used to compare the networks using Welsh’s unequal variances t-test. Networks were visualized and betweenness centrality calculated using Gephi (Bastian et al., 2009).

## 3 Results

### 3.1 The impact of probiotic supplementation on the response of the viable microbiota to antibiotics

**Figure 2:**
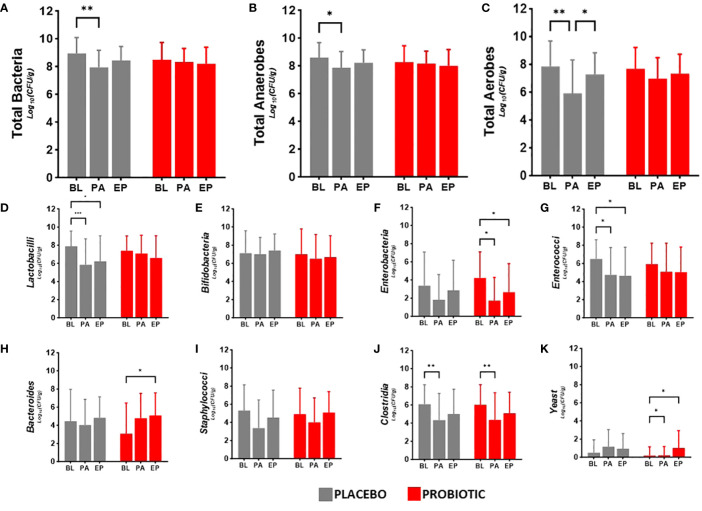
Viable numbers of **(A)** Total Bacteria, **(B)** Total Anaerobes, **(C)** Total Aerobes, **(D)** Lactobacilli, **(E)** Bifidobacteria, **(F)** Enterobacteria, **(G)** Enterococci, **(H)** Bacteroides, **(I)** Staphylococci, **(J)** Clostridia and **(K)** Yeast in faeces at baseline (BL), after antibiotic treatment (post antibiotic, PA) and after the follow-up period (endpoint, EP). Data is presented as the mean log_10_(CFU/gram of faeces) ± standard deviation of 25 participants per group. Values of p were determined mixed-effects analysis with a Tukey’s *post-hoc* for multiple comparisons where *p<0.05, **p<0.01 or ***p<0.001 for within group differences.

Faecal viable numbers are presented in **Figure 2**: total bacteria (**Figure 2A**), total anaerobes (**Figure 2B**), total aerobes (**Figure 2C**), *Lactobacilli* (**Figure 2D**), *Bifidobacteria* (**Figure 2E**), *Enterobacteria* (**Figure 2F**), *Enterococci* (**Figure 2G**), *Bacteroides* (**Figure 2H**), *Staphylococci* (**Figure 2I**), Clostridia (**Figure 2J**) and yeast (**Figure 2K**).

Between group analysis showed no significant differences in the microbiota at each time point. Within group analysis for the probiotic group indicated decreases in the numbers of *Enterobacteria* post antibiotic (−2.50 log_10_CFU/g, p = 0.0197, **Figure 2F**) and between baseline and the end of the study (−1.57 log_10_CFU/g, p = 0.0156, **Figure 2F**), increased *Bacteroides* numbers in the re-growth population (−1.72 log_10_CFU/g, p = 0.0104, **Figure 2H**) with an antibiotic impact on the clostridial numbers (−1.77 log_10_CFU/g, p = 0.0096, **Figure 2J**); yeast numbers significantly increased from baseline after antibiotics (0.91 log_10_CFU/g, p = 0.0282) and at end of the study (0.84 log_10_CFU/g, p = 0.0479). In the placebo group there were significant reductions in the viable numbers of total bacteria (−0.99 log_10_CFU/g, p = 0.0022, **Figure 2A**), total anaerobes (−0.73 log_10_CFU/g, p = 0.0241, **Figure 2B**), aerobes (−1.94 log_10_CFU/g, p = 0.0017, **Figure 2C**) and *Lactobacilli* (−2.05 log_10_CFU/g, p = 0.0009, **Figure 2D**), alongside antibiotic associated decreases in *Enterococci* (−1.75 log_10_CFU/g, p = 0.0302, **Figure 2G**) and Clostridia (−1.69 log_10_CFU/g, p = 0.0034, **Figure 2J**) although numbers had returned to baseline levels by the end of the study. Bifidobacterial numbers did not change in either group during the study (**Figure 2E**) and there were no changes in Staphylococcal numbers in either group over the duration of the study (**Figure 2I**).

### 3.2 Impact of probiotic supplementation on bacterial diversities following antibiotic therapy

**Figure 3:**
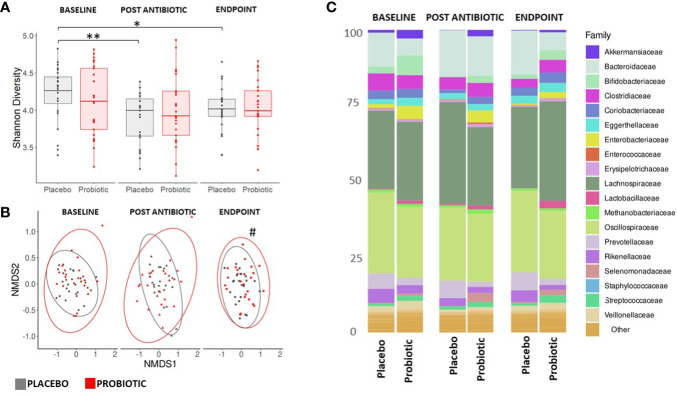
Measures of composition and diversity indices. **(A)** Shannon alpha diversity measures of bacterial taxa and **(B)** NMDS plot displaying bacterial spatial separation by timepoint. **(C)** Composition of gut microbiome at family level. For **(A, C)** values of p were determined by GLM where *p<0.05 or **p<0.01 for within group differences. For **(B)** values of p were determined through PERMANOVA where ^#^p<0.05 for between group differences. BL, baseline; PA, post antibiotic; EP, endpoint.

Faecal samples were extracted and sequenced using a shotgun metagenomics approach on an Illumina NovaSeq 6000 platform. Each sample was subsequently assembled *de novo*, with the resulting number of contigs ranging from 143,121 to 615,638 and used to classify bacterial and fungal taxonomic composition. Alpha diversity indices (Shannon and Simpson) of the microbiota (**Figure 3A**) displayed no changes within the probiotic group between timepoints; whereas there was a significant drop in Shannon alpha diversity in the placebo group between baseline and post antibiotic treatment (p = 0.0320) and between baseline and the end of the study (p = 0.0014, **Figure 3A**). When groups were sub-grouped based on antibiotics administered into β-lactams and macrolides, the Shannon alpha diversity scores significantly decreased at the end of the study compared to the baseline in the placebo group (p = 0.0444 and p = 0.0352, respectively) but no changes were observed in the probiotic group for either antibiotic class (**Supplementary Figure S2**).

Analysis of beta diversity (**Figure 3B**) showed no significant between group spatial separation at the baseline or post antibiotic treatment, however, after re-growth a significant spatial separation was observed between the endpoints of the probiotic and placebo groups (p = 0.0109, **Figure 3B**). There were no significant within group changes observed. Analysis of homogeneity of dispersion showed no differences either between or within groups (data not shown). No differences were observed in either the alpha or beta diversity measures for the mycobiome at any stage of the study (**Supplementary Figure S3**).

### 3.3 Probiotic supplementation and taxonomic changes within the gut microbiota

Bacterial reads accounted for approximately 95% of taxa observed, with Viruses, Archaea and Eukaryota making up approximately 5% with Bacillota, Bacteroidota and Actinomycetota dominant in all participants throughout the study (**Supplementary Figure S4**). At baseline, the microbiota of the probiotic group had a significantly higher proportion of *Enterobacteriaceae* (p = 0.0125), *Akkermansiaceae* (p = 0.0247) and *Bifidobacteriaceae* (p = 0.0089) than the placebo group. Whereas, the placebo group had a higher proportion of *Bacteroidaceae* (p = 0.0001) than the probiotic group.

Post antibiotic treatment, *Akkermansiaceae* and *Bifidobacteriaceae* dropped significantly in both the probiotic (p = 0.0034 and p = 0.0012, respectively) and the placebo (p = 0.0437 and p = 0.0135, respectively) groups. In the placebo group, compared to baseline, the populations of *Enterobacteriaceae* and *Veillonellaceae* decreased following antibiotic treatment (p = 0.0255 and p = 0.0012 respectively) and remained suppressed through the re-growth period (p = 0.0491 and p = 0.0357, respectively). No changes occurred in the *Veillonellaceae* population in the probiotic group (**Figure 3C**). By the end of the study, in the probiotic group the *Enterobacteriaceae* were significantly lower than at the baseline (p = 0.0226), while the *Bifidobacteriaceae* had recovered (p = 0.0410) from the post antibiotic treatment levels back to levels comparable to baseline (**Figure 3C**).

Between group analysis of differentially abundant taxa at the species level at the end of the study indicated higher levels in the placebo group of species belonging to *Enterobacteriaceae* and *Bacteroidiaceae*, including *E. coli* (p < 0.0001)*, Klebsiella pneumoniae* (p = 0.0084) and *Bacteroides fragilis* (p = 0.0021), In the probiotic group, species belonging to *Lactobacillaceae* and *Bifidobacteriaceae* (including *Lactobacillus acidophilus* (p = 0.0003) and *Bifidobacterium longum* (p < 0.0001) increased post-antibiotic treatment and at were higher at the end of the study than at baseline (**Supplementary Figure S5**).

### 3.4 Antimicrobial resistome changes following antibiotic and probiotic treatment

**Figure 4:**
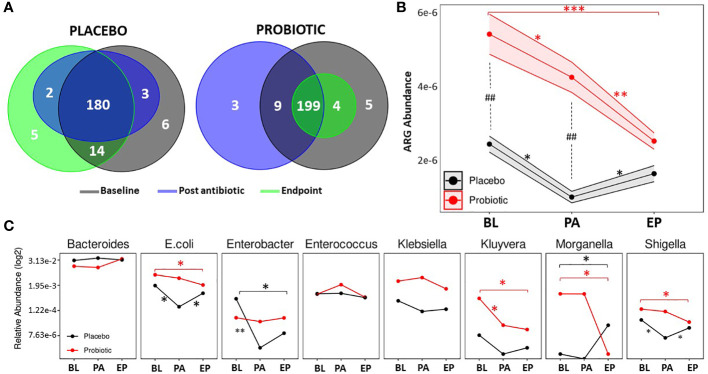
Antibiotic resistant gene analysis. **(A)** Venn diagrams displaying the total number of unique ARGs found at each timepoint in the placebo group and the probiotic group. **(B)** Changes in the relative abundance of total ARGs over the duration of the study in the probiotic and placebo group. **(C)** Changes in the relative abundance of a selection of bacteria containing ARGs over the duration of the study in the probiotic and placebo group. For B and C, values of p were determined by GLM where *p<0.05, **p<0.01 or ***p<0.001 for within group differences and ^##^p <0.01 for between group differences. BL, baseline; PA, post antibiotic; EP, endpoint.

ARGs were detected through the use of the Resistance gene identifier from CARD. In total, 218 ARGs were detected (**Figure 4A**), putatively conferring resistances against antimicrobial classes including aminoglycosides, β-lactams, macrolides, fluoroquinolones, tetracyclines and glycopeptides. At the baseline, 217 ARGs were found in the probiotic group, while 203 ARGs were present in the placebo. Post antibiotic treatment, both groups saw a decrease in overall gene content with 211 present in the probiotic and 185 in the placebo, however, at the end of the study the overall gene content in the probiotic group continued to fall to 203, while the placebo group increased to 201 ARGs. Genes that conferred resistance to tetracycline were amongst the most prevalent in both the placebo and probiotic group, with *tetW* being the overall most abundant ARG.

Between group differences in the total abundance of ARGs were assessed (**Figure 4B**), and showed that the probiotic group had a significantly higher abundance of ARGs compared the placebo group at the baseline (p = 0.0038) and post antibiotic treatment (p = 0.0012) but at the end of the study, there were no significant differences between groups. Within group analysis (**Figure 4B**) found that the overall abundance of ARGs significantly dropped in both groups post antibiotic treatment (p = 0.0125 for the placebo and p = 0.0482 for the probiotic). ARG abundance in the re-growth population of the probiotic group decreased to lower than that post antibiotic (p = 0.0086) and was lower than baseline (p = 0.0002). The ARG abundance for the re-growth population increased in the placebo group (p = 0.0325), with final levels comparable to baseline (p = 0.5235). When the abundance of ARGs was normalized to each group respective baseline, a significant difference (p=0.0340) was found between groups at the end of the study (**Supplementary Figure S6**)

Analysis of individual ARGs between and within group are presented in **Supplementary Tables S2** and **S3**. Interestingly, there were no increases in ARG abundance within the probiotic group during the study, with genes including *rpoB* (p = 0.0095)*, acrA* (p = 0.0064)*, mdtE* (p = 0.0010), *mdtF* (p = 0.0069) and *yojl* (p = 0.0033), significantly decreased from the baseline to the end of the study along with *DHA-16* (p = 0.0001) and *CTX-M-95* (p = 0.0001) being absent in the probiotic group at the end of the study. In contrast, in the placebo group, *VanE* (p = 0.0030)*, tetW* (p = 0.0333)*, ErmT* (p = 0.0111), had increased in abundance by the end of study compared to baseline levels.

The relative abundances of bacterial taxa known to harbour relevant ARGs was assessed (**Figure 4C**). Within the probiotic group, *Kluyvera* decreased between baseline and post antibiotic treatment, whereas *E. coli, Enterobacter* and *Shigella* decreased in the placebo during the same period (**Figure 4C**). Post antibiotic treatment, no changes in microbial abundance occurred in the probiotic group, while *E. coli* and *Shigella* were found to be increased in the placebo group at the end of the study. By the end of the study there were significant decreases in *E. coli*, *Shigella*, *Kluyvera* and *Morganella* in the probiotic group compared to the baseline (**Figure 4C**), whereas, *Enterobacter* decreased after the antibiotic treatment in the placebo group. At the end of the study, *Morganella* was higher than at baseline (**Figure 4C**). Full details of all bacterial taxa linked to ARGs are presented in **Supplementary Figure S7**.

### 3.5 Co-occurrence of bacterial taxa following antibiotic therapy

**Figure 5:**
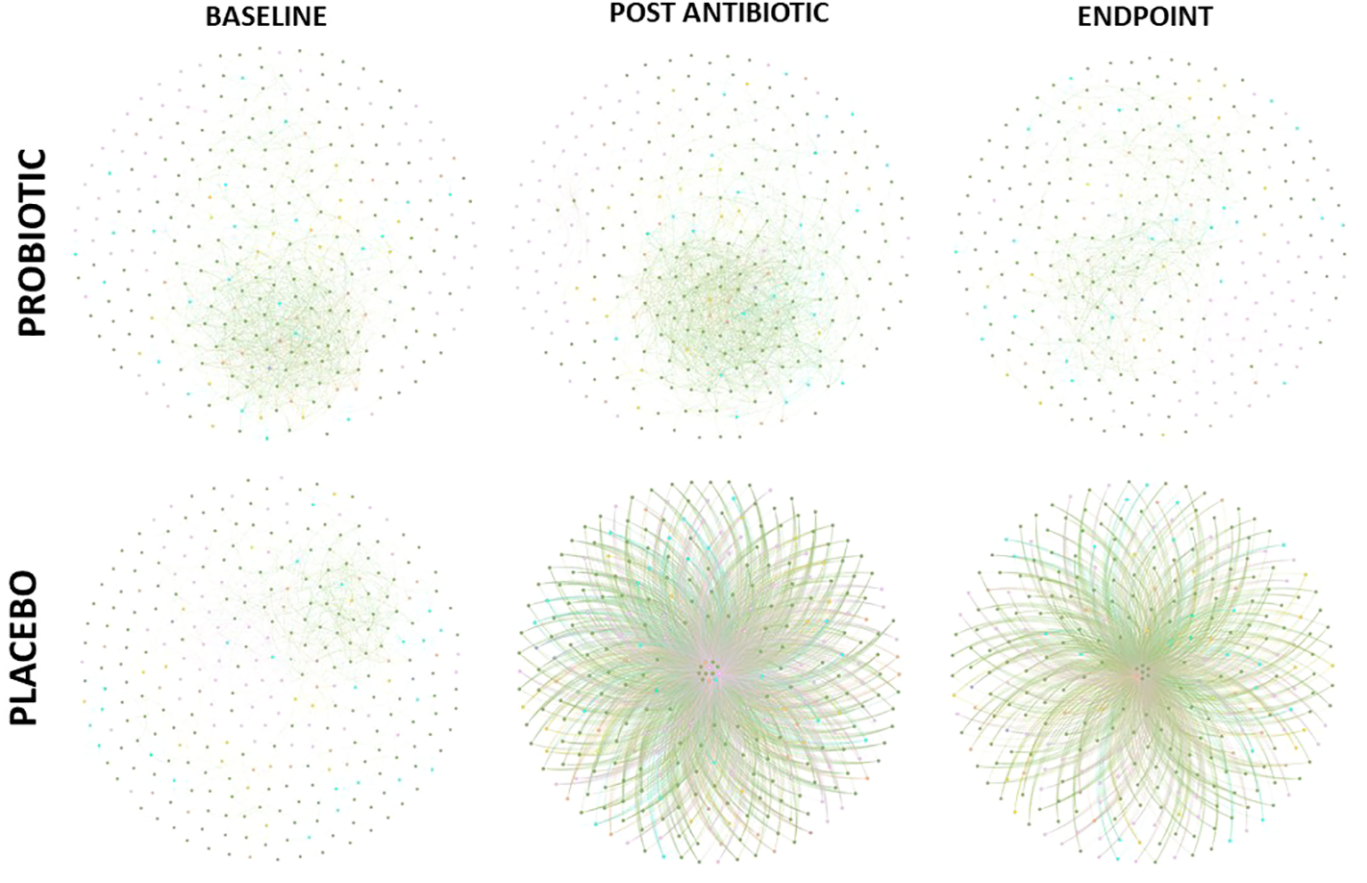
Co-occurrence network of correlations between bacterial composition at genus level (FDR cut off – 0.05). Edges between nodes represented a correlation between taxa.

Co-occurrence networks of bacterial taxa were generated for each group, at each timepoint based on correlation relationships (and FDR adjusted q-values with a cut off of 0.05, **Figure 5**). At the baseline, both groups had similar betweenness centrality and node degree scores despite the different abundances observed, however significant differences were observed post antibiotic (q = 0.0411) and at the end of the study (q = 0.0284). Node degree and betweenness centralities decreased in the placebo group from the baseline to post antibiotic treatment (q = 0.0321) and to the end of the study (q = 0.0105), whereas, the node degree and betweenness centrality scores remained consistent throughout in the probiotic group with no significant differences observed. Keystone analysis at baseline highlighted *Faecalibacterium, Latilactobacillus, Limosilactobacillus* and *Butyricicoccus*, in both groups. These organisms continued as keystone taxa within the probiotic group throughout the study, while in the placebo group *Coprococcus, Clostridium* and *Eggerthella*, were the keystone taxa identified both post antibiotic treatment and at the end of the study.

## 4 Discussion

This exploratory study shows the diversity of the gut microbiota in participants receiving daily supplementation of a probiotic alongside their prescribed antibiotic therapy was not only maintained but was also associated with an overall decrease in the total abundance of ARGs in the re-growth population compared to baseline levels.

Antibiotic treatment is known to negatively affect the gut microbiota causing decreases to both the bacterial number and the alpha diversity scores (Bich et al., 2022). In this study the placebo group followed this recognized pattern with significant decreases in the total numbers of viable bacteria (particularly Lactobacilli, Enterococci and Clostridia) in response to antibiotic treatment. Whereas, in the probiotic group, despite the antibiotic treatment, the numbers remained largely consistent – although the numbers of Enterobacteria were significantly reduced at the end of the study compared to the baseline. Enterobacteria species are widely recognized as carriers of ARGs and can be considered as opportunistic pathogens potentially representing a large threat and so this reduction in numbers could be considered beneficial within individuals taking the probiotic (Mancuso et al., 2021). For those receiving the probiotic the number of yeasts had increased significantly during the study which may reflect the presence of the *Saccharomyces boulardii* in the probiotic product. In the placebo group, there was a significant decrease in the Shannon and Simpson diversity indices post antibiotic treatment that remained consistent to the end of the study. Within the probiotic group, there was no loss of alpha diversity suggesting that the probiotic afforded a protective effect on overall alpha diversity. A meta-analysis investigating the effects of probiotics during and post antibiotic treatment did not always support protective effects on diversity indices (Éliás et al., 2023), however the high variability of the studies means more research with similar probiotic strains and antibiotic types and durations is needed. By the end of the study, there was a significant spatial separation between the probiotic and placebo group which may provide further support for the probiotic playing a role in supporting the composition of the microbiota and this preservation of alpha diversity scores and reduced changes to gut microbial composition has been seen previously (Fernández-Alonso et al., 2022). The presence of *Saccharomyces boulardii* alongside the lactobacilli and bifidobacteria did not appear to have an observable impact on the alpha or beta diversities of the mycobiome. It has been found that antibiotics can have varying effects on the mycobiome, with increased competition between fungal species (Seelbinder et al., 2020; Spatz et al., 2023).

Following antibiotic treatment, the total number of ARGs had decreased in both groups. As the study progressed, the abundance of ARGs within the placebo group started to return to baseline levels whereas, the opposite was seen in the probiotic group where the ARG abundance was found to decrease. It has been in observed in non-probiotic studies that the composition of both the gut microbiota and the ARG content can return to pre-antibiotic levels within one to two months of antibiotic treatment (Palleja et al., 2018; Ng et al., 2019; Anthony et al., 2022). The ARG increase observed in the placebo group suggests the same effect from post antibiotic treatment to the end of the study, while the continued decline in ARG abundance within the probiotic group suggests the probiotic had exerted an effect within the gut. A change in the re-growth population related to the probiotic supplementation is indicated by the change in beta diversities between groups at the end of the study. Post antibiotic treatment, the regrowth population has been observed to differ compared to the original microbiome population (Palleja et al., 2018) and so the probiotics may encourage a more beneficial re-growth population with potentially less ARGs – as shown in this study with stable alpha diversity indices and a lower abundance of ARGs compared to the baseline in the probiotic group.

Assessment of the abundances of bacteria known to carry ARGs found levels of *E. coli*, *Kluyvera* and *Morganella* decreased within the probiotic group. *E. coli* represents one of the most important bacterial populations within the antimicrobial resistance spectrum due to the ability to acquire ARGs through horizontal gene transfer, with the indigenous *E. coli* population being seen as a risk due to transferable plasmid mediated antimicrobial resistance (Tawfick et al., 2022). The significant decrease of *E. coli* observed in the probiotic group could represent a means of achieving a lasting protective effect within the gut microbiota by reducing transfer of plasmid ARGs. The decreases seen in the probiotic group in the populations of *Morganella* and *Kluyvera* may be related to the decrease and/or absence of DHA-16 and CTX-M-95 (two β-lactamases) at the end of the study suggesting that they may have become suppressed in the re-growth population. At the end of the recovery period in the placebo group, differential abundance of the bacterial populations showed increases in *E. coli* and *Klebsiella* which are linked to several antibiotic resistant genes (Ballén et al., 2021; Pakbin et al., 2021; Worby et al., 2023), including *mdtE* and *mdtF;* these ARGs were decreased in the probiotic group at the endpoint, and so could be the drivers of the results observed.

Co-occurrence network analysis of bacterial taxa provided further indications of the potential protective effects achieved by supplementation with probiotics. The probiotic group remained consistent throughout the study with little change in its keystone taxa, such as *Faecalibacterium* and *Lactobacillus* spp., from baseline to endpoint supporting lower antibiotic disruption of the microbiota population and taxa which are linked to benefits within the host (Gardiner et al., 2015; Jiang et al., 2021). Within the placebo group, node degree and betweenness centrality analysis revealed a small number of keystone bacterial taxa, including *Coprococcus, Clostridium* and *Eggerthella*, having potentially more control over functionality of the gut microbiome following antibiotic treatment. *Eggerthella* sp., such as *Eggerthella lenta*, are emerging pathogens which can cause serious intra-abdominal pain and its prevalence as a keystone taxon post antibiotic treatment could cause disruption within the gut (Gardiner et al., 2015; Jiang et al., 2021).

This study has a number of strengths: (i) the use of both traditional culture and metagenomics to comprehensively assess the impact of probiotic supplementation on faecal microbiota composition and ARG content and (ii) findings that support those of a previous study demonstrating the ability of a related probiotic to reduce the extent of gut microbiota disruption and to reduce the level of antibiotic resistance within the “re-growth” microbiota (Plummer et al., 2005). Limitations of the study are; (i) the variability of antibiotic dosage and duration administered to participants (ii) the between group differences in faecal microbiota composition and ARG content at baseline, (iii) the short post antibiotic treatment recovery period, and (iv) the exploratory (unpowered) nature of the study. Large variations in the abundance of ARGs from person to person and different geographic locations have been reported (Fredriksen et al., 2023; Lee et al., 2023), which suggests that an adequately powered follow-up to this exploratory study is needed to better account for person to person and geographic differences in ARG abundance.

In summary, this exploratory, randomized, double-blind, placebo-controlled study identified potential benefits of daily supplementation with the Lab4 probiotic + *Saccharomyces boulardii* when administered alongside antibiotic treatment to reduce antibiotic associated disturbances on the gut microbiome by protecting against loss of diversity, and potentially reducing the level of antibiotic resistance capacity in the re-growth population.

## Data availability statement

The datasets presented in this study can be found in online repositories. The names of the repository/repositories and accession number(s) can be found below: https://www.ebi.ac.uk/ena, PRJEB71357.
