# Corrigendum: A double-blind, randomized, placebo-controlled study assessing the impact of probiotic supplementation on antibiotic induced changes in the gut microbiome

**DOI:** 10.3389/frmbi.2024.1484878

**Published:** 2024-09-05

**Authors:** Daniel John, Daryn Michael, Maya Dabcheva, Eleri Hulme, Julio Illanes, Tom Webberley, Duolao Wang, Sue Plummer

**Affiliations:** ^1^ Research & Development, Cultech Ltd, Port Talbot, United Kingdom; ^2^ Clinical Research Unit, Comac Medical, Sofia, Bulgaria; ^3^ Department of Clinical Sciences, Liverpool School of Tropical Medicine, Liverpool, United Kingdom

**Keywords:** probiotics, microbiome, antibiotics, antibiotic resistance, metagenomics

In the published article, there was an error in the legend for **Supplementary Figure S6** as published. The legend incorrectly mentioned a “selection of bacteria”. The corrected legend appears below.

“**Figure S6**. Changes from baseline in the relative abundance of ARGs over the duration of the study in the probiotic and placebo group. Values of p were determined by GLM where *p<0.05, **p<0.01 and ***p<0.001 for within group differences and #p<0.05, ##p <0.01 and ###p<0.001 for between group differences. Abbreviations: BL, baseline; PA, post antibiotic; EP, endpoint.”

The authors apologize for this error and state that this does not change the scientific conclusions of the article in any way. The original article has been updated.

