# Supplementary material for: A double-blind, randomized, placebo-controlled study assessing the impact of probiotic supplementation on antibiotic induced changes in the gut microbiome
Source: Front Microbiomes. 2024 Mar 22;3:1359580. doi: 10.3389/frmbi.2024.1359580 (PMC12993619; doi:10.3389/frmbi.2024.1359580)
Supplement: Supplementary file 1 [file DataSheet1.docx]

Supplementary Material

**Faecal Sample Collection Instructions:**

**STEP 1:** Label the FANTIB labelled clear-plastic GENbag with your participant number, the date and the time of collection.


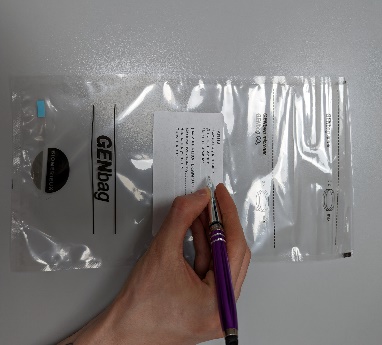


**STEP 2:** Put on the gloves and place the Fe-col faecal collection paper over the toilet seat according to the instructions provided. Sit on the toilet and catch the faecal sample on the collection paper. **Try to avoid wetting the paper.**

**STEP 3:** Open the faecal sample pot and, using the sampling spoon, half fill the plastic pot with faecal sample and close the pot tightly.

**STEP 4:** Flush the Fe-col® collection paper down the toilet.


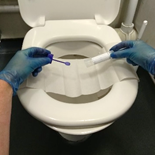

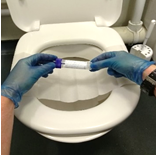

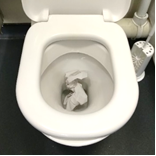


**STEP 5:** Place the faecal sample pot in the FANTIB GENbag. Dispose of gloves and immediately proceed to next step.


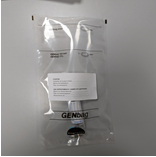


**STEP 6:** Open the GENbag foil sachet, remove the white sachet and immediately place the white sachet in the FANTIB GENbag making sure that the blue plastic strip remains within the bag.


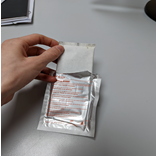

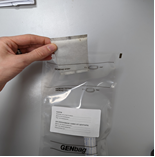


**STEP 7:** Place the grey sealer at the top of the FANTIB GENbag and completely seal by inserting the black cylinder into the grey clip.


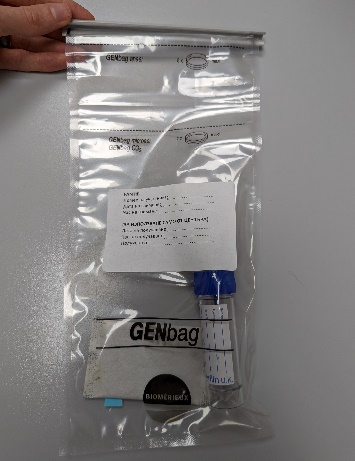

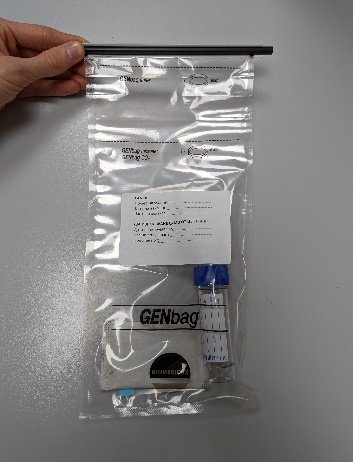


**STEP 8:** Place the sealed and labelled FANTIB GENbag into the white plastic envelope provided and seal the envelope by removing the adhesive strip protector and folding the envelope lid.

**STEP 9:** Store in the sealed envelope in a cool place (<10^o^C) until returned to the trial centre.
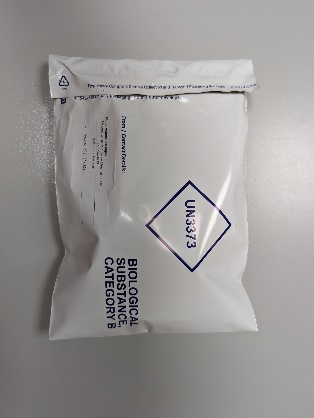


**Figure S1.** Faecal collection instructions provided to participants during the study.


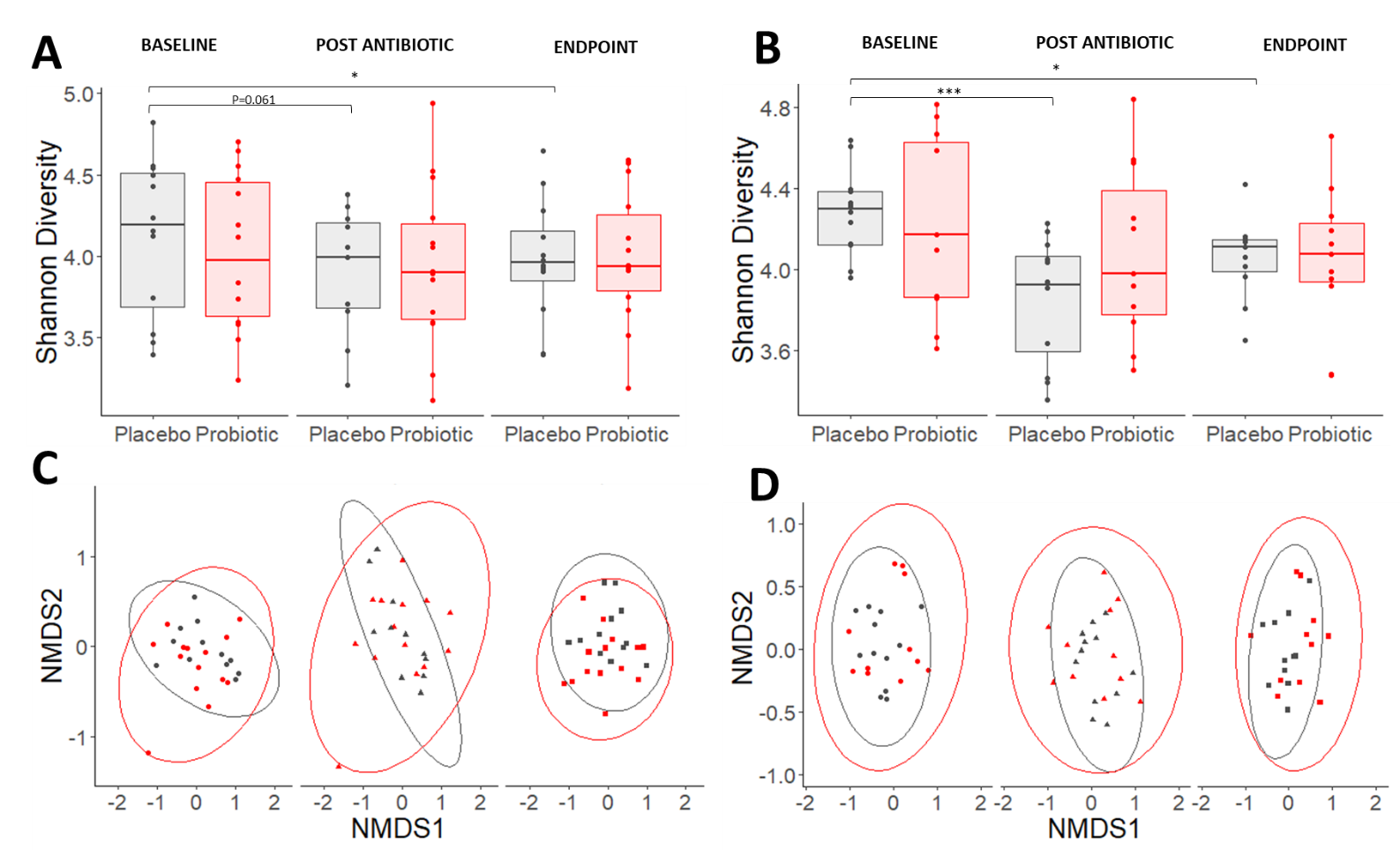


Figure S2. Sub-group analysis of bacterial diversities by antibiotic type administered. (A, C) Shannon diversity plot and NMDS plot of participants given Beta-Lactam antibiotics, (B, D) Shannon diversity plot and NMDS plot of participants given Macrolide antibiotics.

**
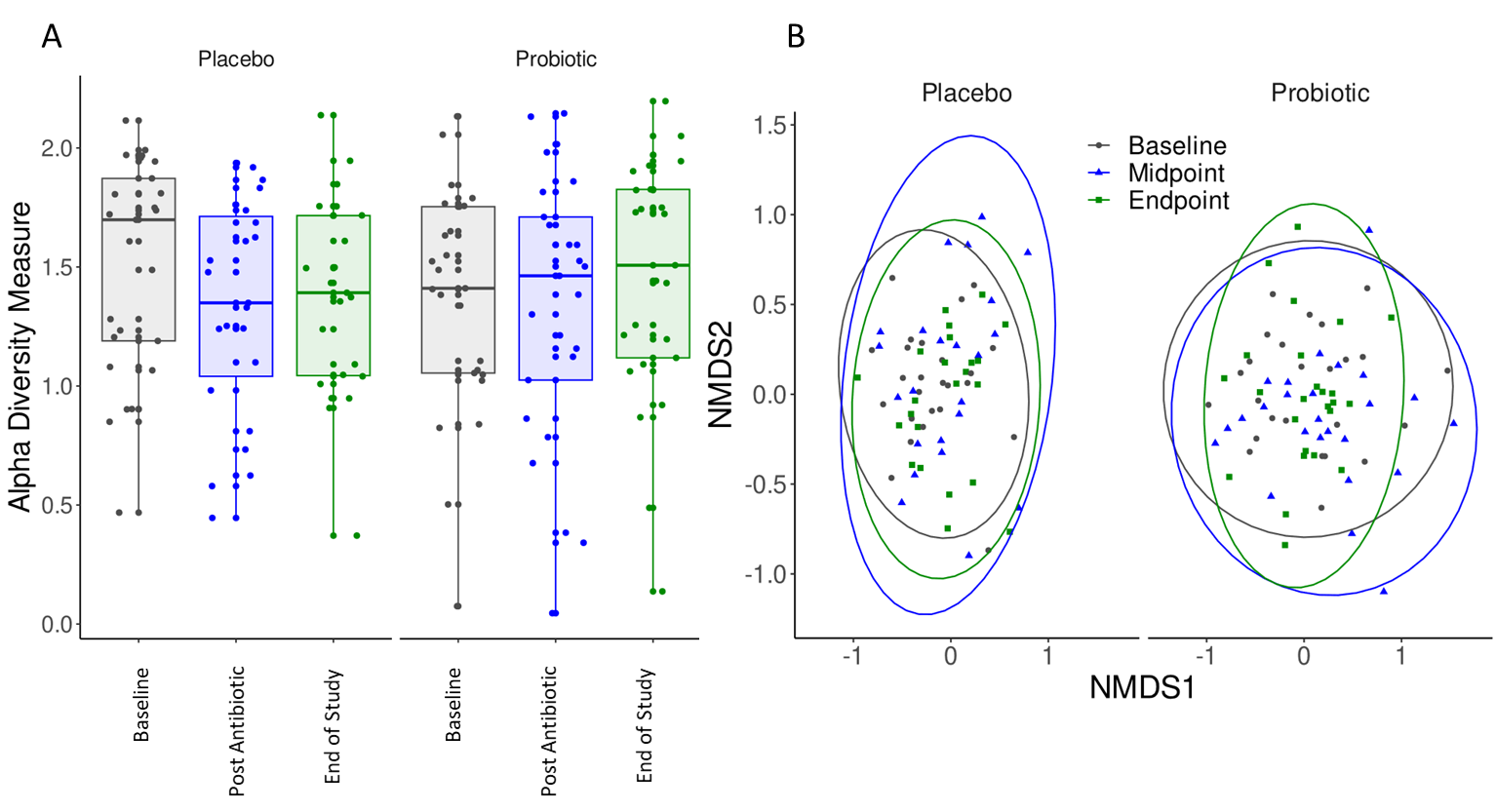
**

Figure S3. Diversity measures of the mycobiome. (A) Shannon alpha diversity and (B) NMDS plot of spatial separation between timepoints in the placebo and probiotic group


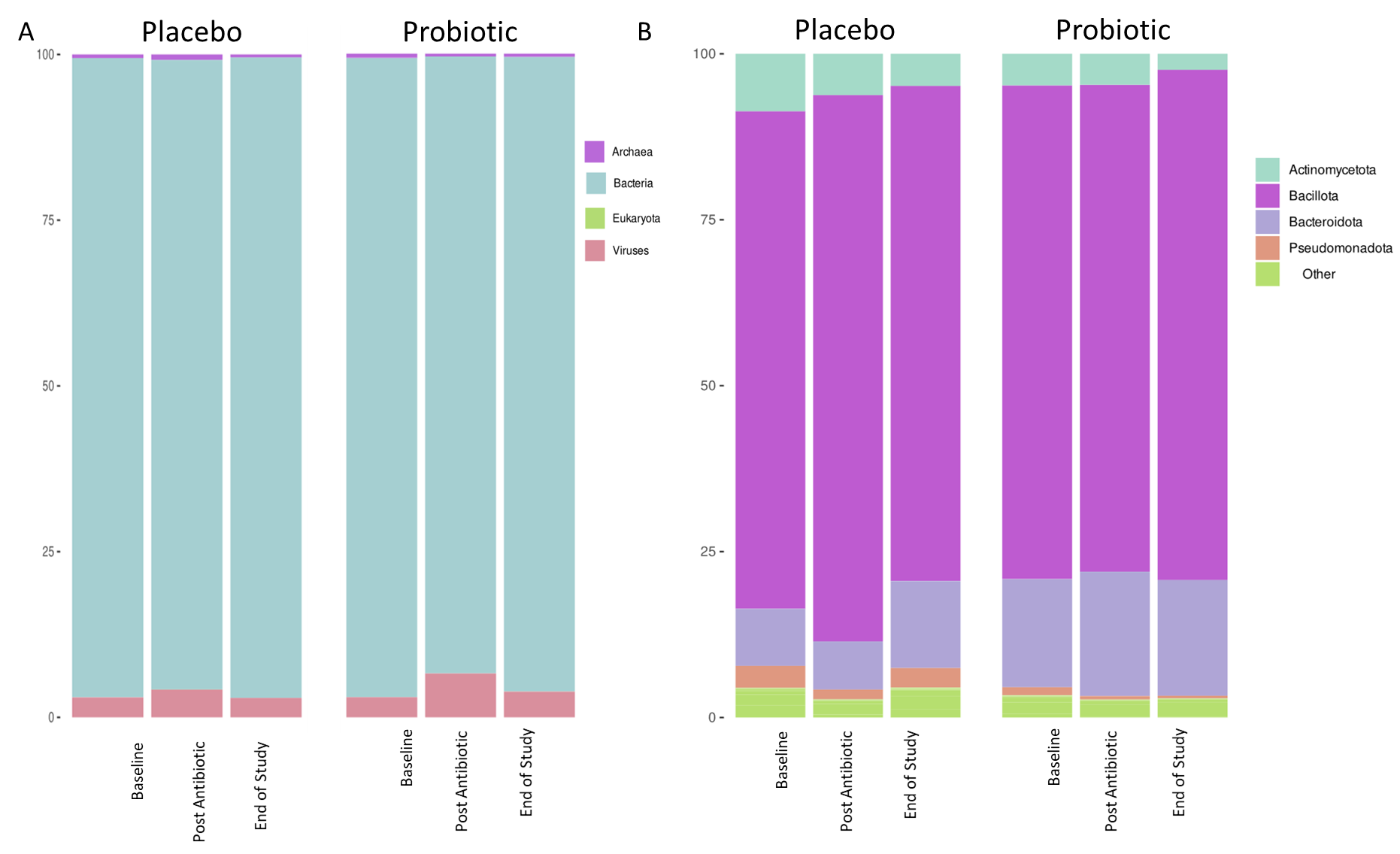


Figure S4. Relative abundance at the (A) Kingdom and (B) Phyla level for all reads identified


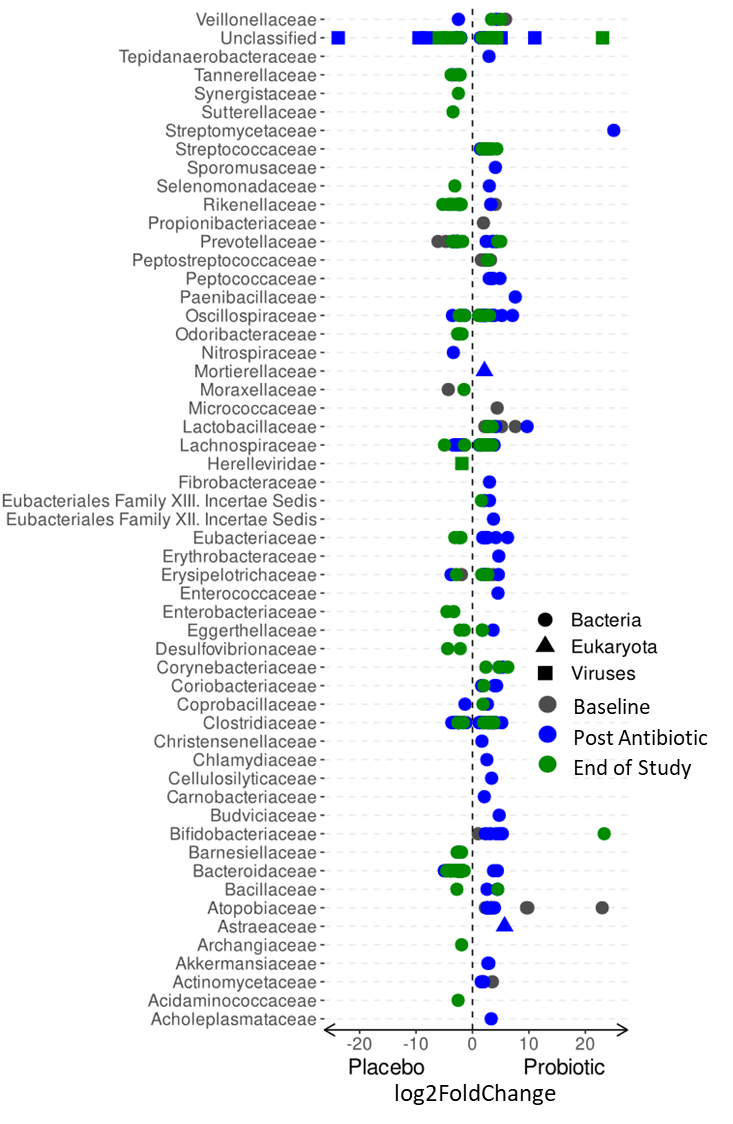


Figure S5. Differentially abundant analysis through DESEQ2 of species found between groups at each timepoint, grouped at family level.

**Figure S6**. Changes from baseline in the relative abundance of ARGs over the duration of the study in the probiotic and placebo group. Values of p were determined by GLM where *p<0.05, **p<0.01 and ***p<0.001 for within group differences and ^#^p<0.05, ^##^p <0.01 and ^###^p<0.001 for between group differences. Abbreviations: BL, baseline; PA, post antibiotic; EP, endpoint


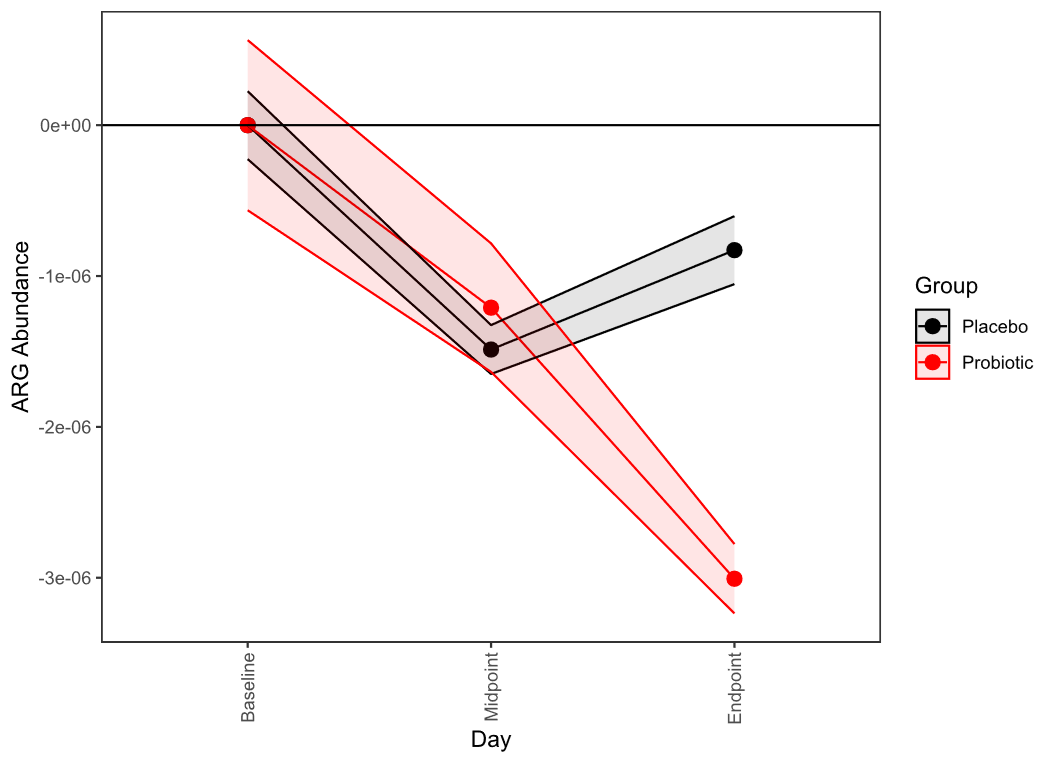

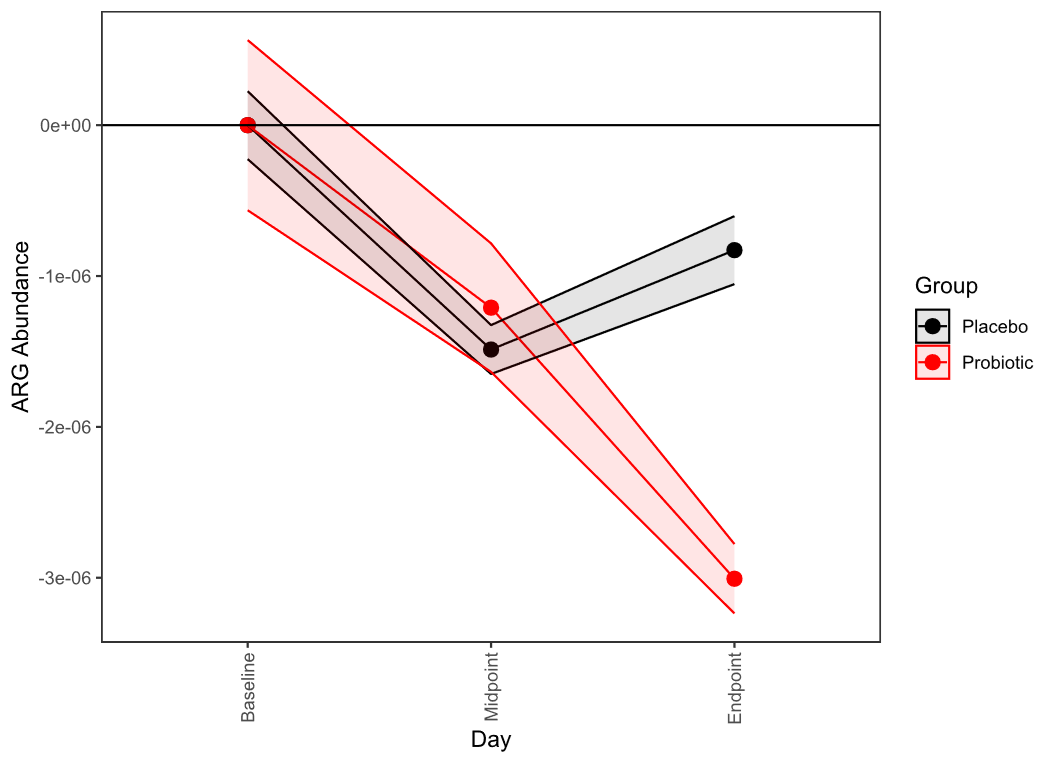


#

*

***

*

*

**BL PA EP**

**
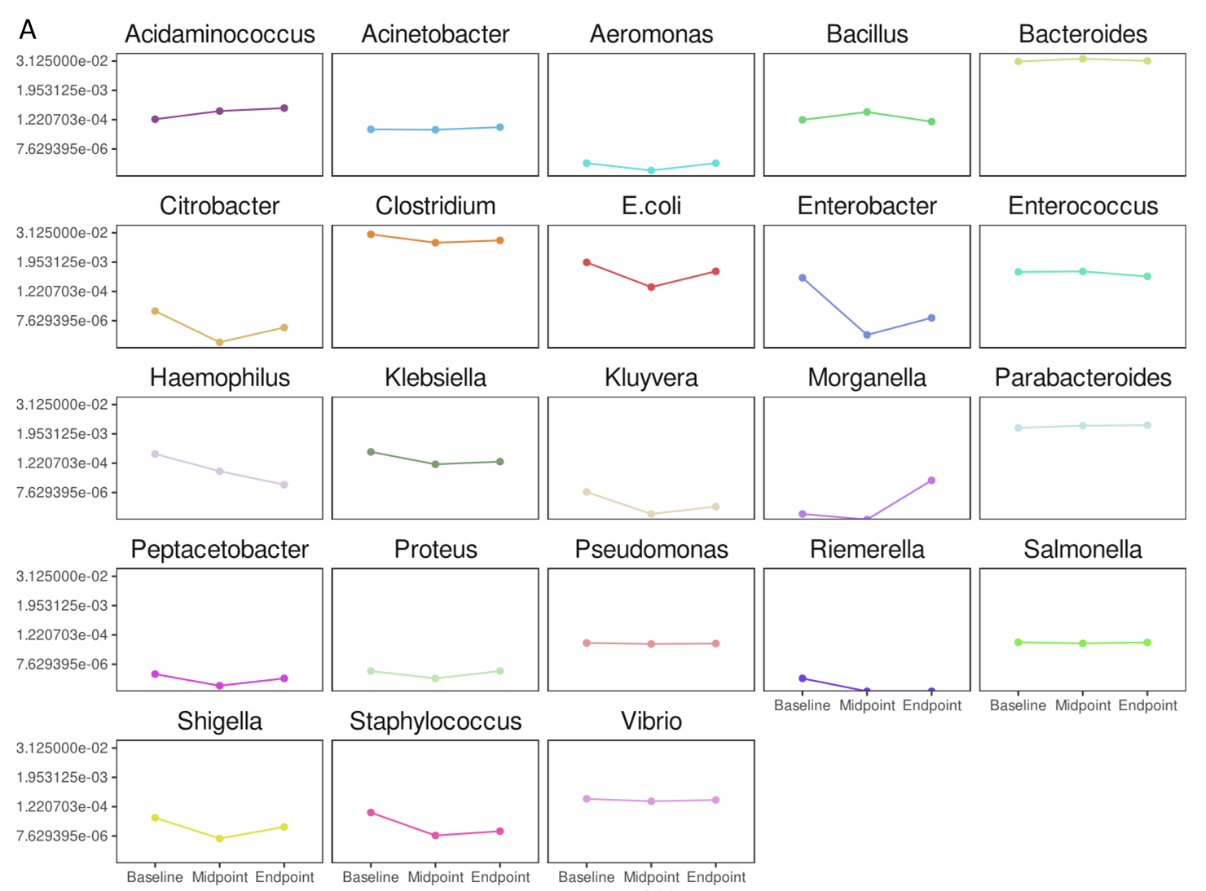
**


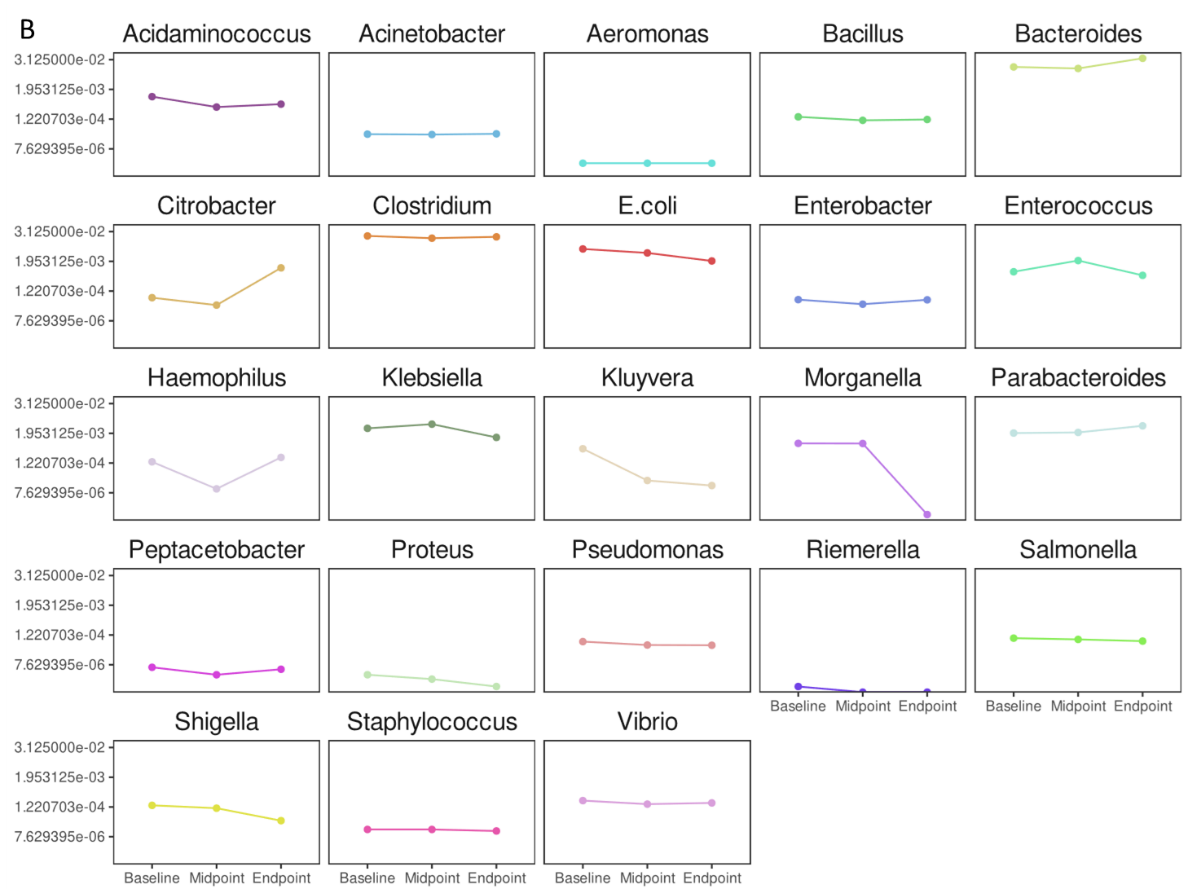


Figure S7. Relative abundance across timepoints for bacterial related to ARGs identified in during the study in the (A) Placebo and (B) Probiotic group.

Table S1. Selective microbial culture conditions. Anaerobic conditions were made with 10% carbon dioxide, 5% hydrogen and 85% nitrogen.

| **Culture** | **Selective medium** | **Growth conditions** |
| --- | --- | --- |
| Lactobacilli | De Man, Rogosa and Sharpe (MRS) agar + Polymixin B (CM0361, SR0099E) | Aerobic/Anaerobic, 37 °C for 48 h |
| Bifidobacteria | MRS-X agar3 + defibrinated sheep blood (SR0051C) | Anaerobic, 37 °C for 48 h |
| Bacteroides | Wilkins- Chalgren Anaerobe agar + G-N Anaerobe Selective supplement (CM0619, SR0108) | Anaerobic, 37 °C for 48 h |
| Enterobacteria | MacConkey No. 3 agar (CM0115B) | Aerobic, 37 °C for 48 h |
| Enterococci | Kanamycin Aesculin Azide agar + Kanamycin supplement (CM0591, SR0092E) | Aerobic, 37 °C for 48 h |
| Clostridia | Horse Blood agar - Shocked with >99.9% ethanol for 30min | Anaerobic, 37 °C for 48 h |
| Staphylocci | Baird Parker agar + egg yolk tellurite emulsion supplement (CM0275B, SR0054) | Aerobic, 37 °C for 48 h |
| Yeast | Dichloran Rose Bengal Chloramphenicol (DRBC) agar + chloramphenicol supplement (CM0727, SR0078E) | Aerobic, 37 °C for 48 h |
| Total Aerobes | Colombia + 5% HBA (Biomerieux, 43050) | Aerobic, 37 °C for 48 h |
| Total Anaerobes | HBA | Anaerobic, 37 °C for 48 h |

Table S2. Table of ARGs with significant increases in abundance across timepoints between group.

| **Gene** | **Description** | **Timepoint** | **Group** | **p value** |
| --- | --- | --- | --- | --- |
| vanXY | antibiotic target alteration | Baseline | Probiotic | 0.0081 |
| rpoB | B subunit for RNA polymerase | Baseline | Probiotic | 0.0082 |
| LptD | antibiotic efflux | Baseline | Probiotic | 0.0116 |
| acrR | antibiotic efflux | Baseline | Probiotic | 0.0116 |
| DHA-16 | DHA Beta-lactamase | Baseline | Probiotic | 0.0213 |
| tetB | antibiotic efflux | Baseline | Probiotic | 0.0229 |
| CfxA6 | antibiotic inactivation | Baseline | Probiotic | 0.0268 |
| VanTr | antibiotic target alteration | Baseline | Probiotic | 0.0438 |
| nimB | antibiotic inactivation | Baseline | Probiotic | 0.0465 |
| ermB | antibiotic target alteration | Baseline | Probiotic | 0.0471 |
| cfrE | antibiotic target alteration | Baseline | Probiotic | 0.0499 |
| rpoB | B subunit for RNA polymerase | Midpoint | Probiotic | <0.0001 |
| SAT-4 | antibiotic inactivation | Midpoint | Probiotic | 0.0016 |
| APH2-Ig | antibiotic inactivation | Midpoint | Probiotic | 0.0071 |
| ermrE | antibiotic target alteration | Midpoint | Probiotic | 0.0303 |
| H-NS | antibiotic efflux | Midpoint | Probiotic | 0.0303 |
| APH3-IIIa | antibiotic inactivation | Midpoint | Probiotic | 0.0326 |
| gadX | antibiotic efflux | Midpoint | Probiotic | 0.0475 |
| pmrF | antibiotic target alteration | Midpoint | Probiotic | 0.0492 |
| rpoB | B subunit for RNA polymerase | Endpoint | Probiotic | 0.0002 |
| SAT-4 | antibiotic inactivation | Endpoint | Placebo | 0.0016 |
| APH2-Ig | antibiotic inactivation | Endpoint | Probiotic | 0.0072 |
| mdeA | antibiotic efflux | Endpoint | Probiotic | 0.0072 |
| soxR | antibiotic efflux | Endpoint | Probiotic | 0.0082 |
| vanU | antibiotic target alteration | Endpoint | Probiotic | 0.0133 |
| ANT6-Ib | antibiotic inactivation | Endpoint | Placebo | 0.0148 |
| adeF | antibiotic efflux | Endpoint | Placebo | 0.0210 |
| DHA-16 | DHA Beta-lactamase | Endpoint | Placebo | 0.0222 |
| tetA46 | antibiotic efflux | Endpoint | Probiotic | 0.0239 |
| tetQ | antibiotic target protection | Endpoint | Placebo | 0.0263 |
| vanH | antibiotic target alteration | Endpoint | Probiotic | 0.0302 |
| emrE | antibiotic efflux | Endpoint | Probiotic | 0.0303 |
| H-NS | antibiotic efflux | Endpoint | Probiotic | 0.0303 |
| vanY | antibiotic target alteration | Endpoint | Probiotic | 0.0326 |
| APH3-IIIa | antibiotic inactivation | Endpoint | Probiotic | 0.0327 |
| cfrE | antibiotic inactivation | Endpoint | Probiotic | 0.0327 |
| efmA | antibiotic efflux | Endpoint | Probiotic | 0.0359 |
| vanT | antibiotic target alteration | Endpoint | Probiotic | 0.0427 |
| nimF | antibiotic inactivation | Endpoint | Probiotic | 0.0473 |
| gadX | antibiotic efflux | Endpoint | Probiotic | 0.0476 |
| PmrF | antibiotic target alteration | Endpoint | Probiotic | 0.0493 |

Table S3. Table of ARGs with significant increases in abundance across timepoints within groups.

| **Gene** | **Description** | **Group** | **Comparison** | **p value** |
| --- | --- | --- | --- | --- |
| VanE | antibiotic target alteration (ligase enzyme) | Placebo | Baseline-Endpoint | 0.0030 |
| TetW | Antibiotic target protection | Placebo | Baseline-Endpoint | 0.0333 |
| EmrB | antibiotic efflux | Placebo | Baseline-Endpoint | 0.0111 |
| mdtP | antibiotic efflux | Placebo | Baseline-Endpoint | 0.0210 |
| emrE | antibiotic efflux | Placebo | Baseline-Endpoint | 0.0011 |
| ermT | antibiotic target alteration (methytransferase) | Placebo | Baseline-Endpoint | 0.0110 |
| mdtE | antibiotic efflux | Placebo | Baseline-Endpoint | 0.0053 |
| AcrF | antibiotic efflux | Placebo | Baseline-Endpoint | 0.0039 |
| SAT-4 | antibiotic inactivation | Placebo | Baseline-Endpoint | 0.0420 |
| rpoB | B subunit for RNA polymerase | Probiotic | Baseline-Endpoint | 0.0095 |
| acrA | protein subunit of acrAB-TolC multidrug efflux pump | Probiotic | Baseline-Endpoint | 0.0064 |
| acrD | aminoglycoside efflux pump | Probiotic | Baseline-Endpoint | 0.0455 |
| acrF | inner membrane transporter | Probiotic | Baseline-Endpoint | 0.0220 |
| cpxA | Stress induced sensor kinase | Probiotic | Baseline-Endpoint | 0.0150 |
| mdfA | Multidrug efflux pump | Probiotic | Baseline-Endpoint | 0.0320 |
| mdtE | Multidrug efflux pump | Probiotic | Baseline-Endpoint | 0.0010 |
| mdtF | Multidrug efflux pump | Probiotic | Baseline-Endpoint | 0.0069 |
| mdtG | Multidrug efflux pump | Probiotic | Baseline-Endpoint | 0.0250 |
| emrY | Multidrug efflux pump | Probiotic | Baseline-Endpoint | 0.0180 |
| yojI | ATP-binding cassette (ABC) efflux pump | Probiotic | Baseline-Endpoint | 0.0033 |
| baeS | Sensor kinase in the BaseSR regulatory system | Probiotic | Baseline-Endpoint | 0.0090 |
| ugd | pmr phosphoethanolamine trasnferae | Probiotic | Baseline-Endpoint | 0.0310 |
| PBP3 | Penicilin binding protein mutations conferring resistance to beta lactams | Probiotic | Baseline-Endpoint | 0.0250 |
| DHA-16 | DHA Beta-lactamase | Probiotic | Baseline-Endpoint | 0.0001 |
| CTX-M-95 | Beta-lactamase | Probiotic | Baseline-Endpoint | 0.0001 |
